# Endothelin Receptor Antagonists for the Treatment of Raynaud's Phenomenon and Digital Ulcers in Systemic Sclerosis

**DOI:** 10.1155/2011/201787

**Published:** 2011-10-27

**Authors:** Kait Arefiev, David F. Fiorentino, Lorinda Chung

**Affiliations:** Division of Immunology and Rheumatology, Departments of Dermatology and Medicine, Stanford University School of Medicine, Palo Alto VA Health Care System, 3801 Miranda Avenue, Palo Alto, CA 94304, USA

## Abstract

Systemic sclerosis is a connective tissue disease characterized by fibrosis of the skin, internal organs, and widespread vasculopathy. Raynaud's phenomenon and digital ulcers are vascular manifestations of this disease and cause significant morbidity. Current treatments are only moderately effective in reducing the severity of Raynaud's in a portion of patients and typically do not lead to substantial benefit in terms of the healing or prevention of digital ulcers. Several studies have evaluated the efficacy of targeting the vasoconstrictor endothelin-1 for the treatment of systemic sclerosis-associated vascular disease. The purpose of this paper is to summarize the published studies and case reports evaluating the efficacy of endothelin receptor antagonists in the treatment of Raynaud's phenomenon and digital ulcers associated with systemic sclerosis.

## 1. Introduction

Systemic sclerosis (SSc) is a connective tissue disease characterized by fibrosis of the skin and internal organs and widespread vasculopathy. Raynaud's phenomenon (RP) is often the first manifestation of SSc, frequently preceding the onset of cutaneous sclerosis by several years particularly in patients with limited disease, and eventually occurs in 95% of patients with SSc [1]. Vasospasm of the digital arteries leads to the three characteristic phases of pallor, cyanosis, then erythema correlating with reduced blood flow, total loss of oxygen supply, and reperfusion. Episodes of RP are usually triggered by cold exposure or stress and can be associated with numbness and pain, resulting in significant disability [2]. Recurrent episodes of ischemia-reperfusion injury and the subsequent generation of reactive oxygen species can result in ischemic damage to distal tissue sites. Digital ulcers (DUs) are necrotic lesions that occur either at distal aspects of digits (fingers or toes) or over bony prominences and occur in up to 50% of patients with limited or diffuse cutaneous SSc [3]. These lesions are exquisitely painful, heal slowly, and interfere with activities of daily living often leading to substantial functional disability. Other complications associated with DU include scarring with loss of distal tissue, infection that can lead to osteomyelitis, and progression to gangrene requiring amputation [4, 5]. DUs that develop at distal aspects of digits are thought to be related to recurrent ischemia from various processes, including vasospasm from RP, thrombosis of digital arteries, calcinosis, and structural microvascular changes related to the underlying SSc [4, 6–8]. Recurrent trauma, particularly in patients with joint contractures, also contributes to the development of DU in patients with SSc. Ulcerations on the lower extremities proximal to the feet can occur in patients with SSc who likely have macrovascular disease as well. Current treatments for both RP and DU consist of vasodilators including calcium channel blockers (CCBs), alpha-adrenergic inhibitors, angiotensin converting enzyme (ACE) inhibitors, angiotensin receptor blockers, and nitroglycerin analogues. These medications are moderately effective in reducing the severity of RP in a portion of SSc patients [9], but typically do not lead to substantial benefit in terms of the healing and prevention of DU. 

With the availability of powerful vasodilator therapies for the treatment of pulmonary arterial hypertension (PAH), options for the treatment of severe RP, DU, and progressive digital ischemia have increased. Prostacyclin analogues have been shown to accelerate the healing of DU, however, those agents found to be effective thus far require intravenous or subcutaneous delivery [10–12]. Small studies have indicated that oral phosphodiesterase-5 inhibitors (PDE-5-I) are effective in reducing the severity of RP and promoting the healing of DU [13–15]. Large multicenter randomized controlled studies are underway to further evaluate the efficacy of PDE-5-I in the treatment of RP and DU. Several studies have evaluated the efficacy of targeting the vasoconstrictor endothelin-1 (ET-1) for the treatment of RP and/or DU. The purpose of this paper is to summarize the published studies evaluating endothelin receptor antagonists (ETRA) in the treatment of RP and/or ischemic DU associated with SSc.

## 2. The Role of Endothelin in the Pathogenesis of SSc-Associated RP and DU

The initial events leading to SSc vasculopathy are thought to involve endothelial cell injury [16] with subsequent loss of normal vasodilatory mediators such as prostacyclin and nitric oxide [17–20]. In addition, endothelial injury results in increased release of the vasoconstrictor endothelin-1 (ET-1) [21, 22]. ET-1 is a 21-amino acid polypeptide expressed primarily by endothelial cells, but has also been found to be expressed by epithelial cells, macrophages, fibroblasts, and cardiomyocytes among others [23, 24]. It acts locally, binding to the surface of smooth muscle cells and acts on the vascular endothelium itself in an autocrine manner. Levels of ET-1 have been found to be increased in the serum of patients with RP and SSc [25–27]. In addition to its role as a biomarker of vascular disease, ET-1 itself may be contributing to the fibrotic and vasculopathic aspects of SSc as it has been shown to stimulate fibroblast and smooth muscle proliferation [28, 29]. ET-1 signaling is mediated by two transmembrane G-protein-coupled receptors (ET_A_ and ET_B_) with different binding affinity and physiologic effects [23, 30]. ET_A_ receptors are expressed on vascular smooth muscle cells and primarily mediate vasoconstriction whereas ET_B_ receptors are expressed on both endothelial cells, mediating vasodilatation, and on smooth muscle cells, mediating vasoconstriction [31].

## 3. Endothelin Receptor Antagonists

Endothelin receptor antagonists are a class of PAH-specific drugs that block the interaction of ET-1 with its receptors (Table 1). ETRAs can selectively act on ET_A _receptors to varying degrees, thus interfering with the vasoconstrictive effects of ET-1. Those with a relatively low ET_A_/ET_B_ selectivity are traditionally considered nonselective. Both nonselective and selective ETRAs have shown efficacy in the treatment of PAH and currently two are approved for this indication in the USA: bosentan and ambrisentan [32–35]. Sitaxsentan was approved in Europe, Canada, and Australia in 2006, but withdrawn from the market in 2010 due to concerns about severe liver toxicity. Case reports of patients showing improvement in their RP and DU while undergoing therapy with ETRAs for PAH have led to randomized controlled trials investigating the efficacy of these agents for the treatment of RP and DU in patients with SSc. As a result of two large randomized controlled trials, bosentan was approved for the prevention of DU in SSc patients in the European Union in June 2007. We will now review the published literature describing the use of ETRAs in the treatment of RP and/or DU in patients with SSc. 

### 3.1. Case Reports

Table 2 summarizes the case reports describing the efficacy of ETRAs in the treatment of SSc-associated cutaneous ulcers. The first case report published in 2003 described a 50-year-old male with diffuse cutaneous SSc and severe PAH who was enrolled in a double-blind, placebo-controlled study investigating the efficacy and safety of bosentan in patients with PAH (the BREATHE-1 study [32, 36]). During the open-label extension phase of the study, he received bosentan 62.5 mg twice daily and within 4 weeks of this therapy, his leg and other small nonacral skin ulcers on his trunk and extremities had healed. In 2006, there were two published case reports describing the efficacy of bosentan in treating cutaneous ulcerations in sclerodermatous conditions. The first case described a 61-year-old female with limited cutaneous SSc and multiple DU refractory to CCB and IV prostacyclin therapy [37]. After 6 months of standard bosentan therapy (62.5 mg  BID × 4  weeks  then  125 mg  BID), she experienced resolution of her DU correlating with a decrease in plasma ET-1 concentration. The second case reported a 4-year-old girl with pansclerotic morphea unresponsive to corticosteroids, methotrexate, CCB, ACE-inhibitors, and D-penicillamine [38]. Within the first months of bosentan therapy, both her widespread sclerotic skin lesions and her limb ulcers improved. Another case report published in 2007 described a 39-year-old female with limited cutaneous SSc and worsening DU despite IV prostacyclin therapy [39]. After 6 weeks of the standard approved dose of bosentan, her DU completely healed. In 2008, a report was published describing a 62-year-old female with long-standing SSc who experienced healing of a large pretibial ulceration after 6 months of standard bosentan therapy [40]. Finally, in 2009, a case report described a 39-year-old female with diffuse cutaneous SSc and recalcitrant DU treated with sitaxsentan 100 mg daily. After 6 months of therapy, her DU significantly improved and no new DU developed [41].

### 3.2. Open-Label Studies

Table 3 outlines the prospective studies investigating the utility of ETRAs in the treatment of RP and/or DU. The first prospective study published in 2006 described 3 patients with RP in the setting of prescleroderma (defined as RP associated with sclerodermatous nailfold capillaroscopic changes and SSc-specific autoantibodies) or limited cutaneous SSc independent of a history of DU [43]. The participants received the standard dosing of bosentan and at the end of the 16-week treatment course pain, RP disease activity and severity were noted to be reduced. A larger prospective observational study published in 2008 evaluated the long-term efficacy and tolerability of bosentan in 15 patients with SSc with current or a prior history of DU [44]. The patient population in the study was particularly heterogeneous with a wide range in age (11–72 years), 0 to 26 DU at baseline, and included 6 patients with interstitial lung disease and 3 with PAH. They were treated with bosentan therapy at standard doses and were followed for a mean of 24.7 months (range 4–36 months). There was a significant decrease in the mean number of DU per patient from 5 at baseline to 0.4 at 12 months (*P* < 0.05). In 2009, an observational study was published on 15 patients with connective tissue disease associated PAH that specifically evaluated the effect of bosentan on DU and RP [45]. After a median of 8 weeks of treatment, 13 out of 15 patients had improved RP severity with 8 patients experiencing disappearance of all RP symptoms after a mean of 14 weeks. Healing of DU was observed after a median of 20 weeks (range 16–24 weeks) for 6 of the 8 patients who had DU at baseline. The longest prospective open label study of bosentan was a 3 year trial of 26 patients with DU refractory to CCB, ACE-inhibitors or sildenafil, published in 2009 [46]. Complete healing of DU occurred in 17 of the 26 participants (65%) after a median period of 25 weeks (range 8–26 weeks), and improvement was noted in the DU of 4 additional patients. Overall, the mean number of DU per patient was reduced at 6, 12, and 36 months (*P* < 0.001). Additionally, healing of the DU was evidenced by complete reepithelialization on skin biopsy which was performed on 5 of the 26 participants. In a 24-week prospective open-label trial, 10 patients with SSc were treated with bosentan; skin fibrosis as assessed by the modified Rodnan skin score (MRSS) was the primary endpoint, but evaluation of DU was a secondary outcome assessment [48]. At each visit, examination of DU was performed by the same evaluator and categorized as present, indeterminate (>50% reduction in their surface area), or healed (total reepithelialization). 88% of DU were categorized as healed at the end of the 24-week treatment compared with 42% at baseline (*P* = 0.0019). Finally, in 2010, a 48-week observational study was published evaluating the effectiveness of bosentan for RP without DU in patients with SSc-associated PAH [49]. 14 patients who were on stable doses of other PAH-specific therapies (excluding patients treated with parenteral prostanoids within the previous 6 months) were treated with bosentan as add-on therapy. For patients with limited or diffuse cutaneous SSc, the number of RP attacks from baseline to 48 weeks decreased from 3.4 ± 1.8 to 2.1 ± 1.4 and 3.8 ± 1.7 to 2.1 ± 1.8, respectively (*P* < 0.05). Likewise, the duration of RP attacks decreased for patients with limited or diffuse cutaneous SSc from baseline to 48 weeks from 62.5 ± 43.7 to 22.1 ± 13.8 minutes and 61.0 ± 38.9 to 29.2 ± 13.7 minutes, respectively (*P* < 0.01).

### 3.3. Double-Blind, Randomized, Placebo-Controlled Studies

The first randomized placebo-controlled double-blind clinical trial evaluating an ETRA for the prevention of DU in patients with SSc was published in 2004 and is commonly known as the RAPIDS-1 (Randomized Placebo-controlled Investigation of Digital ulcers in Scleroderma) trial [42]. 122 patients with SSc and current DU or a history of at least 1 in the prior 12 months, enrolled across 17 centers in Europe and North America. 79 were randomized to receive bosentan and 43 to receive matching placebo during the 16-week treatment phase. The mean number of new ulcers during the treatment period was 1.4 for patients on bosentan versus 2.7 for patients on placebo (*P* = 0.0083) representing a 48% reduction in the number of new DU. However, there were no differences in the reduction of preexisting DU in the 63% of patients with active DUs at baseline. The most notable adverse event occurring in more patients on bosentan than placebo was elevated transaminase levels (14% versus 0%). 3 patients developed a marked transaminitis (>8x ULN), and 5 patients (6%) discontinued the study due to these laboratory abnormalities, but in all cases the transaminase values returned to normal when bosentan was discontinued. The results of a second randomized placebo-controlled double-blind clinical trial investigating bosentan for the treatment of SSc-related DU (RAPIDS-2) were recently published in 2011 [50]. This trial involved a longer 24-week treatment phase and all 188 patients enrolled across 41 sites in Europe and North America were required to have at least 1 active DU, the largest called the “cardinal ulcer,” at baseline. The mean number of new DU over 24 weeks was 1.9 ± 0.2 for patients on bosentan versus 2.7 ± 0.3 for patients on placebo (*P* = 0.035) representing a 30% reduction in the number of new lesions. As with the RAPIDS-1 study, the RAPIDS-2 study failed to show a benefit in terms of healing of existing ulcers. Adverse events occurring in more patients on bosentan than placebo included peripheral edema (18.8% versus 4.4%) and elevated aminotransferases (12.5% versus 2.2%). Markedly increased aminotransferases (>3x upper limit of normal (ULN) and 1 case of >8x ULN) occurred in 10.5% of patients in the bosentan group, but these abnormalities resolved during continued treatment, after a decrease in dose, or following temporary or permanent treatment discontinuation. Only one placebo-controlled double-blind clinical trial evaluating an ETRA for the treatment of RP in patients with SSc has been published to date [47]. 17 patients without preexisting DU were randomized to either standard bosentan therapy or matching placebo during the 16-week treatment phase. Severity of RP was assessed via the Raynaud's Condition Score (RCS), a validated composite self-assessment of the severity of RP encompassing the number and duration of episodes, the associated symptoms, such as pain and numbness, and the degree of hand disability. The RCS is measured on a scale of 0–10 with 0 indicating no disability related to RP and 10 indicating extremely severe disability from RP. Patients recorded the frequency, duration, and severity of RP attacks in daily symptom diaries. The mean RCS score was reduced for both the bosentan and placebo groups (−31% and −36%) at week 16, but the improvements were not statistically significant compared with baseline nor were they different between the groups. Frequency of RP attacks significantly decreased in the bosentan and placebo groups, however, at week 16 only patients in the placebo group maintained a statistically significant decrease in RP frequency (bosentan: −30%; placebo: −57%, *P* = 0.017). In 16 weeks, a significant reduction in mean duration of RP attacks was observed for both bosentan (−26%; *P* = 0.012) and placebo (−60%; *P* = 0.028) compared with baseline, however, there was no significant difference between the groups. Interestingly, despite the lack of improvement on measures of RP activity and severity with bosentan, patients in the treatment group demonstrated statistically significant improvements in functional status as assessed by the scleroderma Health Assessment Questionnaire (*P* = 0.03 and *P* = 0.01 at weeks 12 and 20, respectively) and the UK functional score (*P* = 0.04 at weeks 8 and 16) compared with those treated with placebo. No serious adverse events were noted, and only 1 participant withdrew due to treatment-related peripheral edema. The authors did mention that DU developed in 1 patient on placebo and 2 on bosentan, but these resolved with similar healing times.

## 4. Conclusion

The findings from the literature reviewed here indicate that ETRAs may play a role in the treatment of RP and DU in addition to their indication for the treatment of PAH. The two large, randomized, placebo-controlled studies using bosentan have shown that this agent is useful in the prevention of new DU in patients with SSc (RAPIDS 1 and 2) confirming findings in uncontrolled observational studies. However, both of these studies failed to show a benefit in terms of healing of existing ulcers [42, 50]. Although observational studies demonstrated an improvement in RP with ETRA treatment [43, 45, 49], the one randomized, placebo-controlled study using bosentan for the treatment of RP [47] did not show a statistically significant difference between bosentan and placebo. Although this was a relatively small study, the results highlight the importance of performing RCT in the assessment of potential treatments for RP and DU. In addition to the benefit of ETRA for DU, 3 of the case reports suggested benefit in nondigital ischemic ulcers in patients with SSc, but larger studies are necessary to verify these results [36, 38, 40]. This is important as nondigital ulcers are seen in up to 4% of patients with SSc [51]. The majority of studies published thus far have described the effects of the ETRA bosentan on RP and/or DU. It is unknown whether ETRAs with greater selectivity for the ET_A_ receptor, would show better tolerability and efficacy than bosentan in the treatment of SSc-associated RP and DU. Ambrisentan may be preferable to bosentan given the lower incidence of liver function test abnormalities, once daily dosing and lack of interaction with warfarin. Our center has recently completed the first open-label prospective study of ambrisentan for the treatment of SSc-associated DU and the results will soon be available. ETRAs may be preferable to prostacyclins given their oral bioavailability, but physicians must be cognizant of their teratogenicity and potential side effects including liver toxicity, edema, and anemia. Additional RCTs are necessary to better assess the role of ETRAs for the treatment of RP and DU in patients with SSc.

## Figures and Tables

**Table 1:** Endothelin receptor antagonists.

Endothelin receptor antagonist	Oral dose	Relative ET_A_/ET_B_ selectivity	Information
Nonselective			
Bosentan	Starting: 62.5 mg twice daily Maintenance: 125 mg twice daily	20x	FDA approved for use in the USA in November 2001 for WHO functional class III/IV PAH then extended to include WHO class II in 2009. Approved in the EU for WHO functional class III PAH in May 2002. In June 2007, the EU approved and extended the indication of bosentan as a therapy to reduce the number of new DU in patients with SSc and ongoing DU disease.

Selective			
Ambrisentan	Starting: 5 mg daily Maintenance: 5 or 10 mg daily	4000x	FDA approved for the once-daily treatment of WHO functional class II/III PAH in June 2007. It was later approved by the European Medicines Agency for the same indication in the EU in April 2008.
Sitaxsentan	100 mg daily	6500x	Approved in the EU in August 2006, then in Canada and Australia in March 2007 for the once-daily treatment of WHO functional class III PAH. On December 10, 2010, the manufacturer voluntarily removed sitaxsentan from the market and halted clinical trials due to concerns about liver toxicity.

FDA: Food and Drug Administration, EU: European Union, PAH: pulmonary arterial hypertension, DU: digital ulcer(s), RP: Raynaud's phenomenon, and SSc: systemic sclerosis.

**Table 2:** Case reports of the efficacy of endothelin receptor antagonists for systemic sclerosis-associated cutaneous ulcers.

Author date	Case report	Location of ischemic ulcer(s)	Prior treatments for SSc and/or ulcer(s)*	Results
Humbert and Cabane 2003 [36]	50-year-old male with diffuse SSc and PAH	Trunk Leg DU	IV prostacyclin	Received bosentan 62.5 mg twice daily and within 4 weeks of this therapy his leg and other small nonacral skin ulcers on his trunk and extremities had healed. After an additional 6 months of 125 mg twice daily, his DU completely healed.

Tillon et al. 2006 [37]	61-year-old female with limited SSc and pulmonary sarcoidosis	DU	CCB IV prostacyclin	Bosentan was initiated at the standard approved dose of 62.5 mg twice daily for a month then 125 mg twice daily. After 6 months, she experienced resolution of her DU correlating with a decrease in plasma ET-1 concentration. No new DU developed.

Roldan et al. 2006 [38]	4-year-old female with pansclerotic morphea	Ankles	Corticosteroids Methotrexate CCB, PUVA ACE-inhibitors D-penicillamine	Bosentan was started at an initial dose of 31.25 mg four times daily for 4 weeks, and then decreased to the standard dose for her weight of 31.25 mg twice daily. Within the first months of bosentan therapy, both her widespread sclerotic skin lesions and her limb ulcers improved.

Chamaillar et al. 2007 [39]	39-year-old female with limited SSc	DU	IV prostacyclin	Bosentan was initiated at the standard approved dose and after 6 weeks of this therapy her DU completely healed. No recurrence was noted over 2 years of continued therapy.

Ferreira and Scheinberg 2008 [40]	62-year-old female with diffuse SSc	Pretibial	CCB Antibiotics Antiplatelets IV prostacyclin Sympathectomy	Bosentan was initiated at the standard approved dose with improvement in the ulcer seen during the first few months of therapy. Complete healing of the large pretibial ulceration occurred after 6 months of therapy.

Gholam et al. 2009 [41]	39-year-old female with diffuse SSc	DU	Methotrexate Corticosteroids Cyclosporine CCB	Treated with sitaxsentan 100 mg daily; during the 6 months of treatment there was a decrease in pain and near complete healing of preexisting DU and no development of new DU.

*Treatments noted in the case report only; other treatments may have been used.

SSc: systemic sclerosis, DU: digital ulcers, PAH: pulmonary arterial hypertension, CCB: calcium channel blocker, ET-1: endothelin-1, ACE: angiotensin converting enzyme, and PUVA: psoralen plus ultraviolet A.

**Table 3:** Studies evaluating efficacy of endothelin receptor antagonists for systemic sclerosis-associated raynaud's phenomenon and/or digital ulcers.

Author date	Study type	Intervention	Patients enrolled/ completed	Duration	Primary endpoint for assessment of RP and/or DU	Results
Korn et al. 2004 *RAPIDS-1 *[42]	R, PC, DB	(a) 62.5 mg bosentan BID × 4 weeks; 125 mg BID × 12 weeks (b) Placebo BID × 16 weeks	(a) 79/66 (b) 43/37	16 weeks	Number of new DU developing during the 16-week study period.	Patients receiving bosentan had a 48% reduction in the mean number of new DU at the end of the treatment period (*P* = 0.0083). No difference between groups in the healing of existing ulcers.

Selenko-Gebaue et al. 2006 [43]	Obs	62.5 mg bosentan BID × 4 weeks; 125 mg BID × 12 weeks	3/3	16 weeks	RP activity and pain severity.	Pain, RP disease activity, number and severity of Raynaud's attacks all decreased.

García dela Peña-Lefebne 2008 [44]	Obs	62.5 mg bosentan BID × 4 weeks; then 125 mg BID	15	4 to 36 months	Number and severity of DU.	There was a decrease in the number of DU. A trend towards efficacy was seen in the number of healed ulcers and in the severity of ulcers.

Funauchi et al. 2009 [45]	Obs	62.5 mg bosentan BID × 4 weeks; then 125 mg BID	15	40 to 96 weeks	Number and severity of DU and frequency and severity of RP.	After a median 8 weeks of treatment, 13 out of 15 patients had improved RP. DU also improved after a median 12 weeks' treatment in all of the 8 patients that had DU.

Tsifetaki et al. 2009 [46]	Obs	62.5 mg bosentan BID × 4 weeks; then 125 mg BID	26/23	36 months	Number of new and healed DU.	The mean number of DU per patient was reduced at 6, 12, and 36 months (*P* < 0.001).

Nguyen et al. 2010 [47]	R, PC, DB	(a) 62.5 mg bosentan BID × 4 weeks; 125 mg BID × 12 weeks (b) Placebo BID × 16 weeks	(a) 9/8 (b) 8/8	16 weeks	RCS, frequency, duration, and pain associated with RP attacks.	Compared with placebo, bosentan did not significantly reduce the severity, frequency, duration, or pain of RP attacks.

Kuhn et al. 2010 [48]	Obs	62.5 mg bosentan BID × 4 weeks; 125 mg BID	10/8	24 weeks	Healing of current DU.	Bosentan increased the number of healed DU from 42% at baseline to 88% at week 24 (*P* < 0.0019).

Giordano et al. 2010 [49]	Obs	62.5 mg bosentan BID × 4 weeks; 125 mg BID	14/14	48 weeks	Number and duration of RP attacks	Number and duration of RP attacks showed a statistically significant decrease at 12 weeks and maintained through 24 and 48 weeks (*P* < 0.05).

Mattuci-Cerinic et al. 2011 *RAPIDS-2 *[50]	R, PC, DB	(a) 62.5 mg bosentan BID × 4 weeks; 125 mg BID × 20 weeks (b) Placebo BID × 24 weeks	(a) 98/75 (b) 90/73	24 weeks	Number of new DU and the time to healing of a preexisting DU.	Bosentan treatment was associated with a 30% reduction in the number of new DU compared with placebo (*P* = 0.04). No difference between groups in healing rate of preexisting ulcers.

R: randomized, PC: placebo controlled, DB: double blind, Obs: observational, DU: digital ulcer, RP: Raynaud's phenomenon, and RCS: Raynaud's Condition Score.
